# Heat of Formation of Beryllium Chloride

**DOI:** 10.6028/jres.065A.004

**Published:** 1961-02-01

**Authors:** Walter H. Johnson, Alexis A. Gilliland

## Abstract

The heat of formation of beryllium chloride has been determined, by the direct combination of the elements in a calorimeter, according to the process,

$$\begin{array}{l} \;{\text{Be}(\text{c})+\text{Cl}}_{2}(g)={\text{BeCl}}_{2}(c),\\ \Delta H{}^{\circ}(25\;{}^{\circ}C)=-493.85\pm 2.35\;\text{kj}/\text{mole},\\ \;\;=-118.03\pm 0.56\;\text{kcal}/\text{mole}. \end{array}$$

The data obtained by other investigators are discussed briefly.

## 1. Introduction

This investigation was undertaken as a part of a program, currently in progress at the National Bureau of Standards, on the thermodynamic properties of light-element compounds.

There are very few data in the literature on the thermochemistry of beryllium halides, probably because of the difficulty in obtaining sufficiently pure samples of beryllium, the toxicity of the material, and its stability toward dry halogens. Beryllium is apparently quite stable with respect to dry chlorine at ordinary temperatures. At temperatures up to 250 °C the amount of reaction during a half-hour exposure to dry chlorine appears to be negligible; at about 350 °C, however, the reaction is spontaneous and rapid.

## 2. Materials

The beryllium was obtained in the form of 80- mesh powder from the Brush Beryllium Co. A complete analysis was furnished with the material; the constituents found to be present in quantities greater than 0.01 percent are as follows:
Be…………99.4BeO…………  0.26C………… .06Al………… .06Fe………… .05F………… .015Si………… .015

The chlorine was obtained from the Matheson Co. and was certified to have a purity of not less than 99.85 percent. It was passed through a column packed with phosphorus pentoxide to remove any traces of moisture.

The helium was obtained from the U.S. Bureau of Mines and was certified to be oil free; it was passed successively through columns packed with Ascarite, magnesium perchlorate, and phosphorus pentoxide to remove traces of carbon dioxide and moisture.

## 3. Apparatus

The glass reaction vessel was similar to that used for measurement of the heat of formation of titanium tetrachloride [l].^1^ The only significant changes involved the use of a 375-ohm manganin heating coil and a plug of Pyrex wool in the top of the vessel to prevent the finely divided beryllium chloride from being carried out of the vessel.

The calorimeter was similar to that described previously [2], except that a smaller calorimeter can and well were used, which resulted in a significantly lower energy equivalent. The calorimeter jacket was maintained at 27 °C to within ±0.001 °C during each experiment by a controller which has been described [2]. The sensing element of the jacket temperature controller was changed prior to these experiments by the substitution of a 575-ohm nickel thermometer for the 28-ohm platinum thermometer formerly used; an equivalent change was made in the corresponding arm of the Wheatstone bridge circuit.

The thermometric system, the apparatus for measurement of electrical energy, and the general calorimetric procedure have been previously described [2, 3].

## 4. Units of Energy, Molecular Weights, and Heat Capacities

The unit of energy is the joule, obtained as the product of absolute amperes, absolute volts, and mean solar seconds. All instruments were calibrated with reference to standards maintained at the National Bureau of Standards. For conversion to the conventional thermochemical calorie, one calorie is taken as 4.1840 joules.

All atomic weights were taken from the 1957 Table of International Atomic Weights [4].

The heat capacities used for correction of the data to 25 °C were taken, where possible, from the available literature [5]. In the case of BeCl_2_, an estimated value of 16.3 cal/deg mole was used for the heat capacity of the crystalline state at 25 °C.

## 5. Procedure

Elaborate precautions were taken to avoid personal contact with the metal or inhalation of the metal dust when loading or cleaning the vessel. A sample (0.2 to 0.5 g) of the metal powder was placed in a quartz crucible and lowered into the reaction vessel. A plug of Pyrex wool was placed in the top of the vessel, the glass joint was lubricated with fluorocarbon grease and the vessel was assembled. The vessel was then transferred to the calorimeter can together with 3740 g water, the calorimeter was assembled, connections between the vessel and the gas train were made, and the calorimeter preheater and platinum resistance thermometer were inserted. Air was removed from the reaction vessel by evacuation; the vessel was then refilled with dry helium to a pressure of 1 atm.

Each calorimetric experiment consisted of three parts, a 20-min initial rating period, a 90-min reaction period, and a final 20-min rating period. Calorimeter temperatures were observed at 1-min intervals during the reaction period and at 2-min intervals during the rating periods. At the start of the reaction period, the vessel was heated electrically and a stream of dry chlorine was introduced. It was necessary to pass approximately 0.25 amp at 99 v through the heating coil to obtain the required temperature of 350 °C. At this temperature, the reaction is nearly self-sustaining and continues for some time after interruption of the heating current. After about 20 min, when the desired temperature rise had been obtained, the electric current was interrupted. The flow of chlorine was continued for an additional 20 min, then stopped, and dry helium was passed through the vessel for 30 min. The flow of helium was then stopped and after an additional 20 min the system again was at thermal equilibrium.

Traces of adsorbed chlorine were removed from the vessel by evacuation through a cold trap; the vessel was refilled with dry helium, removed from the calorimeter, and transferred to a well-ventilated hood for analysis of the contents.

Moist air was passed for 2 hrs through the vessel and through two successive scrubbing columns containing about 100 ml of water each. Water was then forced through the vessel and the remaining material washed into the scrubbing columns. The amount of reaction was determined by the concentration of chloride ion in the resulting solution. This determination was made gravimetrically by precipitation with silver nitrate. Determination of the amount of hydrochloric acid by titration of the resulting solution with standard alkali was also made; this determination was less precise, however, probably because of the presence of beryllium hydroxide.

No attempt was made to weigh the amount of unreacted beryllium metal, because of the difficulty of quantitative removal from the vessel and because of the reaction between the metal and the strongly acidic solution formed by hydrolysis of the beryllium chloride.

The quantities of helium and of excess chlorine were determined from the time of flow and the rates of flow as indicated on calibrated capillary flow-meters. The temperatures of the helium and chlorine which entered the system were taken as equal to that of the calorimeter jacket through which they passed. The temperatures of the exit gases were taken as equal to the mean calorimeter temperature during the time of flow. These data were required only for the small gas-temperature correction in which an error of 10 percent would not be significant.

The calorimetric system was calibrated in the same maimer except that no sample was included and only helium was passed through the vessel during the ‘‘reaction” period.

## 6. Results and Calculations

The results of the calibration experiments are given in table 1, where Δ*Rc* is the corrected temperature rise of the calorimetric system [6] expressed as ohms increase in resistance of the platinum resistance thermometer, *E* is the quantity of electrical energy introduced [2, 3], *q_g_* is a correction for the energy carried into the system by the helium, Δ*e* is the deviation in the heat capacity of the actual system from that of the selected “standard” system, and *E_s_* is the energy equivalent of the “standard” calorimetric system.

The results of the calorimetric reaction experiments are given in table 2. In this table, the quantity *q_g_* includes the necessary corrections to convert the reactants and products to their thermodynamic Standard states at 25 °C. The results of the experiments correspond to the reaction:

$$\begin{array}{l} \;{\text{Be}(\text{c})+\text{Cl}}_{2}(g)={\text{BeCl}}_{2}(c),\\ \Delta H{}^{\circ}(25\;{}^{\circ}C)=-493.85\pm 2.35\;\text{kj}/\text{mole},\\ \;\;=-118.03\pm 0.56\;\text{kcal}/\text{mole}. \end{array}$$

No correction was made for the effect of the impurities in the beryllium. Since the quantity of reaction was based on the amount of chloride in the solution of the reaction products, the error would be limited to the difference between the heats of reaction of chlorine with beryllium and with the impurities. We have assumed that BeO would not react with chlorine under our experimental conditions and have calculated the combined corrections for the other materials to be negligible in comparison with other experimental errors.

The uncertainty interval has been taken as twice the standard deviation of the mean for the calibration and reaction experiments, combined with 0.26 percent for the determination of the amount of reaction, 0.01 percent for the determination of the gas heat-capacity correction, 0.10 percent for the effect of impurities, and 0.01 percent for errors in calibration of measuring instruments.

## 7. Discussion

Mielenz and Von Wartenberg [7] obtained −112.6 kcal/mole for the heat of formation of BeCl_2_ by igniting a sample of beryllium in a stream of chlorine gas. They also obtained −135.9 kcal/mole for the heat of formation of BeO by combustion of the metal in an oxygen bomb. Cosgrove and Snyder [8] have reported −143.1 kcal/mole for the heat of formation of BeO, an increase of 5 percent over the value obtained by Mielenz and Von Wartenberg. If this difference is due to the presence of an inert impurity it would seem quite possible that their value for BeCl_2_ is also low by a similar amount. An increase of 5 percent in their value for BeCl_2_ gives −118.2 kcal/mole for the heat of formation which is in good agreement with the result of this investigation.

Siemonsen [9] measured the heat of reaction of beryllium with liquid chlorine in a bomb, reporting −109.2 kcal/mole for the heat of formation of BeCl_2_(c). This value appears to be low and may be due to a systematic error in the calculation or interpretation of his results. His value of −94.8 kcal/mole for the heat of formation of LiCl(c) [10], which appears to be low by about 3 kcal/mole, would indicate that some systematic error might be involved.

Rossini, Wagman, Evans, Levine, and Jaffe [5] gave −122.3 kcal/mole as a selected “best” value for the heat of formation of BeCl_2_(c), based on −146.0 kcal/mole for the heat of formation of BeO(c). A recalculation of their data, using −143.1 kcal/mole for the heat of formation of BeO(c), as determined by Cosgrove and Snyder, gives −119.4 kcal/mole for the heat of formation of BeCl_2_(c) which is in reasonably good agreement with the results of this investigation.

## Figures and Tables

**Table 1:** Results of the calibration experiments

Experiment	Δ*Rc*	*E*	*q_g_*	Δ*e*	*E_8_*
					
	*Ohm*	*j*	*j*	*j/ohm*	*j/ohm*
1	0.217132	35129.4	15.3	−7	161852
2	.224491	36331.1	8.3	−7	161868
3	.205927	33310.1	9.2	−7	161795
4	.238395	38578.1	7.8	−7	161850
5	.251439	40691.8	6.6	−7	161855
	
Mean					161844
Standard deviation of the mean					±13

**Table 2:** Results of the experiments on the heat of formation of beryllium chloride

Experiment	Δ*Rc*	Δ*e*	*E*	*q_g_*	BeCl_2_	−ΔH° (25 °C)
						
	*Ohm*	*j/ohm*	*j*	*j*	*Mole*	*kj/mole*
1	0.251393	13.3	29321.8	3.3	0.022990	494.62
2	.217197	9.2	33137.9	−10.7	.004044	495.90
3	.266267	14.2	31987.2	−3.5	.022359	496.75
4	.238121	10.3	31081.8	−5.5	.015146	492.12
5	.257638	11.1	32384.8	−4.2	.018904	492.54
6	.238719	12.5	29247.5	−7.0	.019174	489. 40
7	.290191	13.9	33312.8	−5.8	.027727	492.34
8	. 267642	11.6	31714.3	−9.2	.023326	497.12
	
Mean						493.85
Standard deviation of the mean						±0.951

## Footnotes

1 Figures in brackets indicate the literature references at the end of this paper.
